# Nonlinear Dose-Response Association Between Stressful Life Events and Probable Problematic Internet Use Among Chinese College Students: Cross-Sectional Study

**DOI:** 10.2196/92310

**Published:** 2026-09-08

**Authors:** Wenxian Zhan, Juan Ge, Yan Wang

**Affiliations:** 1 School of Nursing and Health Management, Shanghai University of Medicine & Health Sciences, Shanghai, China; 2 College of Health Management, Shanghai Jian Qiao University, No. 1111 Hucheng Ring Road, Pudong New Area, Shanghai, 201306, China, 86 021 68192015

**Keywords:** stressful life events, internet addiction, cross-sectional study, nonlinear relationship, restricted cubic spline

## Abstract

**Background:**

Problematic internet use (PIU) is an important public health concern among college students because of its associations with adverse psychological, social, and academic outcomes. Stressful life events may contribute to PIU, but previous studies have mainly relied on linear models and have rarely examined nonlinear, threshold, or plateau effects while accounting for sociodemographic and lifestyle factors.

**Objective:**

This study aimed to examine the linear and nonlinear dose-response associations between stressful life–event scores and probable PIU among Chinese college students, and to determine whether these associations remained after adjustment for potential confounders.

**Methods:**

This cross-sectional study used convenience sampling to recruit students from 8 higher education institutions, including comprehensive universities, medical universities, and vocational colleges, in Shanghai, Jiangsu, and Zhejiang, China, between March 1 and June 30, 2025. Stressful life events during the preceding 12 months were assessed using the 26-item Adolescent Self-Rating Life Events Checklist (ASLEC). Probable PIU was screened using the 8-item Internet Addiction Diagnostic Questionnaire (IADQ), with a score of 5 and above indicating probable PIU. Descriptive statistics, independent-samples 2-tailed *t* tests, chi-square tests, multivariable logistic regression, and restricted cubic spline (RCS) analyses were conducted. The primary analyses used a complete-case sample of 2805 participants.

**Results:**

Among the 2805 participants, 436 (15.5%, 95% CI 14.3%‐16.9%) met the screening criterion for probable PIU. Participants with probable PIU had higher mean ASLEC scores than those without probable PIU (30.26, SD 28.10 vs 17.75, SD 20.84; *P*<.001). In the fully adjusted model, each 1-point increase in the ASLEC score was associated with 2% higher odds of probable PIU (adjusted odds ratio 1.02, 95% CI 1.01‐1.02; *P*<.001). RCS analysis showed significant overall and nonlinear associations (both *P*<.001). The estimated odds changed little below a score of approximately 14, increased markedly between approximately 14 and 60, and appeared to plateau above 60. Because relatively few participants scored above 60, estimates in this range were less precise.

**Conclusions:**

This study is innovative in applying RCS analysis to characterize the nonlinear dose-response association between stressful life–event scores and probable PIU. Unlike previous studies based mainly on conventional linear models, it identified potential inflection points at approximate scores of 14 and 60 and provided a more detailed account of how the odds of probable PIU varied across exposure levels. These findings extend the existing evidence and may provide preliminary information for developing tiered screening, stress-management support, and referral pathways in university mental health services. However, the identified values are exploratory statistical points rather than validated clinical cutoffs. Longitudinal and intervention studies are needed to confirm their practical use and determine whether stress-reduction and coping-focused interventions can reduce the onset or persistence of probable PIU.

## Introduction

### Problem

In the digital era, the internet has become an indispensable tool for daily life, academic pursuits, and social interaction. This is particularly true for college students, who are navigating a critical developmental period characterized by heightened neuroplasticity, identity formation, and social role transition [1,2]. The internet provides an accessible and relatively low-risk environment for identity exploration, emotional expression, and social connection, which may be particularly appealing to students navigating this developmental stage [3]. College students may therefore be particularly susceptible to problematic internet use (PIU), especially in the context of increased autonomy from parental supervision, heightened academic demands, and the expansion of both online and offline social networks [4,5]. Meanwhile, the widespread availability of mobile internet services and the diversification of online applications have further increased college students’ reliance on the internet for learning, communication, and recreation [6]. Despite its academic and social benefits, the widespread use of the internet also raises concerns about problematic use patterns, particularly among college students who are at a developmentally vulnerable stage.

The public health significance of PIU among college students is underscored by its prevalence and associations with a range of adverse health and functional outcomes [7]. A previous systematic review reported a pooled prevalence of 41.84% for internet addiction, as defined in the included original studies, among university students [8]. However, prevalence estimates vary substantially across studies because of differences in assessment instruments, cutoff criteria, operational definitions, sampling strategies, cultural and regional contexts, and periods of data collection [9-11]. Therefore, pooled estimates should not be directly compared with proportions derived from a specific screening instrument and population. Despite this methodological heterogeneity, PIU has consistently been associated with depression, anxiety, loneliness, reduced subjective well-being, lower resilience, sleep disturbances, suicidal ideation, poorer academic achievement, impaired attention, and reduced real-world social functioning [12-16]. Collectively, these adverse outcomes may compromise college students’ psychological well-being, academic development, and social functioning, highlighting the importance of identifying potentially modifiable factors associated with PIU.

### Review of Relevant Scholarship

Among these factors, stressful life events refer to adverse or demanding experiences that require psychological or behavioral adaptation and may exceed an individual’s available coping resources [17]. Such events represent an important source of psychological distress among college students and may arise across multiple domains [18-20]. Academic stressors, including examination failure, heavy study demands, and educational pressure, may contribute to emotional exhaustion and impaired self-regulation [21]. Interpersonal difficulties, such as peer conflict, discrimination, romantic problems, and strained relationships with teachers, may increase loneliness and negative affect [22]. Family conflict, parental pressure, financial hardship, serious illness, bereavement, punishment, and other adverse experiences may further undermine perceived control and psychological well-being [23]. Although these events differ in content, they may contribute to PIU through shared pathways involving psychological distress, maladaptive coping, and impaired self-regulation [15,16]. Individuals experiencing stressful events may use the internet to avoid real-life stressors or regulate negative emotions, thereby increasing their susceptibility to PIU.

Previous studies have reported associations between stressful life events and PIU [24-27]; however, they have generally relied on conventional linear modeling approaches. For example, Yang et al [27] used logistic regression without examining possible nonlinear, threshold, or saturation effects. Consequently, the dose-response association between stressful life events and PIU remains insufficiently characterized. A nonlinear association is theoretically plausible, as low levels of stress may be manageable, moderate levels may overwhelm coping resources and promote maladaptive internet use, and very high levels may be accompanied by emotional exhaustion or co-occurring psychological problems that alter the association. In addition, previous studies have not always adequately considered potential sociodemographic and lifestyle confounders. Age, sex, ethnicity, only-child status, living arrangement, accommodation type, alcohol consumption, breakfast frequency, and outdoor activity may be associated with psychological distress, exposure to stressful life events, or patterns of internet use [28-30] and were therefore considered when estimating the association between stressful life events and PIU.

### Hypotheses, Aims, and Objectives

Accordingly, the primary aim of this study was to examine the dose-response association between stressful life–event scores and probable PIU among Chinese college students, with particular attention to potential nonlinearity. The secondary aim was to evaluate whether this association remained after adjustment for potential sociodemographic and lifestyle confounders. We hypothesized that higher stressful life–event scores would be associated with greater odds of probable PIU and that the association would exhibit a nonlinear pattern.

## Methods

### Conditions and Design

This study used a nonexperimental, observational, cross-sectional design. No intervention, experimental manipulation, or random allocation was involved. The study was conducted from March 1, 2025, to June 30, 2025, among college students enrolled at 8 higher education institutions in Shanghai; Jiangsu Province; and Zhejiang Province, China. The participating institutions included comprehensive universities, medical universities, and vocational colleges and were selected based on existing collaborative relationships with the research team and their willingness to participate.

### Inclusion and Exclusion

Eligible participants were college students enrolled at one of the participating institutions who were able to understand the questionnaire and voluntarily agreed to participate. Participants were excluded from the final analysis if they had missing data on stressful life events, the Internet Addiction Diagnostic Questionnaire (IADQ) [31], or one or more prespecified sociodemographic or lifestyle covariates required for the complete-case analysis.

### Participant Characteristics

The study population consisted of college students recruited from 8 higher education institutions in eastern China. Participant characteristics included age, sex, ethnicity, domicile, only-child status, accommodation type, living arrangement, current smoking, current alcohol consumption, breakfast frequency, and daily outdoor activity time.

### Sampling Procedures

Participants were recruited using convenience sampling. Investigators who received standardized training distributed the electronic questionnaire link through class-based WeChat (Tencent) groups at the participating institutions and invited eligible students to participate voluntarily. A total of 3200 students were invited, of whom 3058 submitted the questionnaire. No financial or material compensation was provided to participants.

### Sample Size, Power, and Precision

No formal a priori sample size or power calculation was performed because this study used a multicenter convenience sample and aimed to recruit all eligible and accessible students during the study period. Of the 3200 students invited, 3058 submitted the questionnaire. The final complete-case analytic sample included 2805 participants, of whom 436 met the screening criterion for probable PIU.

### Measures and Covariates

#### Stressful Life Events

The Adolescent Self-Rating Life Events Checklist (ASLEC) was used to assess the occurrence and perceived impact of stressful life events over a 12-month recall period [32]. The version used in this study included 26 scored items and one open-ended “other event” item, which was excluded from calculation of the total score. The 26 scored items were grouped into 5 dimensions: interpersonal relationships (items 1, 2, 4, and 15), academic stress (items 3, 9, 22, and 25), punishment (items 18‐21, 23, 24, and 26), loss (items 11‐14, 16, and 17), and adaptation problems (items 5‐8 and 10). For each item, participants indicated whether the event had occurred during the preceding 12 months. Events that had not occurred were scored 0. For events that had occurred, participants rated the perceived psychological impact on a 5-point scale ranging from 1 (no impact) to 5 (extremely severe impact). Item scores were summed to obtain a total score ranging from 0 to 130, with higher scores indicating a greater cumulative impact of stressful life events. The ASLEC captures event occurrence and perceived impact but does not assess the precise timing, frequency, duration, or recurrence of each event and therefore cannot distinguish acute from recurrent or chronic stressors. In the original validation study, the scale demonstrated a Cronbach α of 0.85 and a split-half reliability coefficient of 0.88 [32].

#### Probable PIU

Probable PIU was assessed using the 8-item IADQ, developed by Young [31,33]. Although the original name of the instrument uses the term “internet addiction,” the IADQ was used in this study as a self-report screening measure of generalized PIU rather than as a clinical diagnostic instrument. The IADQ was adapted from the diagnostic criteria for pathological gambling and assesses addiction-like features of PIU, including loss of control, tolerance, withdrawal-like symptoms, and impairment in social, academic, or occupational functioning [31]. Representative items include “extending the time spent online beyond the original intention,” “repeated unsuccessful attempts to regulate or diminish internet usage,” “experiencing irritability or restlessness during attempts to curtail internet usage,” and “endangering or forfeiting a significant relationship, employment, or educational opportunity because of internet use.” Each item was answered dichotomously, with “yes” scored as 1 and “no” scored as 0, yielding a total score ranging from 0 to 8. Participants with a total score of ≥5 were classified as meeting the screening criterion for probable PIU. A preliminary reliability and validity study of the Chinese self-report version reported good internal consistency, with a Cronbach α of 0.817 [34].

#### Participant Characteristics and Covariates

A structured questionnaire was used to collect participant characteristics and potential confounding variables. Sociodemographic variables included sex, age, ethnicity, domicile, only-child status, accommodation type (on-campus vs off-campus), and living arrangement. Health-related lifestyle variables included current smoking, current alcohol consumption, breakfast frequency, and daily outdoor activity time [4,35,36]. Breakfast frequency was included as a potential lifestyle-related confounder because it may reflect the regularity of daily routines and broader health-related behavior patterns and has been associated with psychological distress and unhealthy lifestyle behaviors among students [37]. Participants who had smoked at least one cigarette during the preceding 30 days were classified as current smokers. Those who had consumed at least one alcoholic drink during the same period were classified as current drinkers. Both variables were coded as yes or no. Breakfast frequency was categorized as daily (7 d/wk), sometimes (1‐6 d/wk), or never (0 d/wk). Daily outdoor activity time was categorized as less than 1 hour, 1 to less than 2 hours, 2 to less than 3 hours, or at 3 hours or more.

### Data Collection

Data were collected using a self-administered electronic questionnaire developed on an online survey platform. Before the questionnaire link was distributed, trained investigators explained the study’s purpose, procedures, the voluntary nature of participation, and the right to withdraw. Participants completed the questionnaire anonymously through the electronic survey platform.

### Quality of Measurements

To enhance measurement quality, all investigators received standardized training before data collection. The study’s purpose, questionnaire administration procedures, and quality-control requirements were explained consistently across the participating institutions. Submitted questionnaires were reviewed promptly by trained quality-control personnel for completeness and logical consistency. When potential errors or inconsistencies were identified, investigators contacted participants for clarification or correction, as necessary and feasible.

### Instrumentation

The ASLEC and IADQ were previously developed self-report instruments and were not created specifically for the present study. Their content, scoring procedures, interpretation, and prior reliability evidence are described in the *Stressful Life Events* and *Probable PIU* sections. A structured questionnaire was used to assess participant characteristics and prespecified sociodemographic and lifestyle covariates.

### Masking

Masking was not applicable because this was a cross-sectional, self-administered questionnaire survey without treatment allocation or investigator-rated outcomes.

### Psychometrics

In the present sample, both instruments demonstrated high internal consistency. Cronbach α was 0.967 for the ASLEC and 0.884 for the IADQ. No additional psychometric analyses were conducted in this study.

### Missing Data

Before analysis, the data were examined for completeness, logical consistency, and coding accuracy. Submitted questionnaires with missing data on the primary exposure, outcome, or one or more prespecified covariates were identified during data cleaning. The distributions of continuous variables were examined using descriptive statistics before inferential analyses.

Of the 3058 respondents, 253 (8.3%) had missing data, including 98 with missing stressful life events data, 64 with missing IADQ data, and 91 with missing data for one or more prespecified covariates. The Missing Completely at Random (MCAR) test developed by Little indicated that the data were not missing completely at random (*χ*²_2_=65.307, *P*<.001). Multiple imputation was considered; however, because the proportion of missing data was relatively limited and the missingness involved the primary exposure, outcome, and several categorical covariates, the primary analyses were conducted using a complete-case approach. The potential bias associated with complete-case analysis was considered when interpreting the findings.

### Analytic Strategy

Continuous variables were presented as means and SDs, and categorical variables were presented as frequencies and percentages. Between-group comparisons were performed using independent-samples 2-tailed *t* tests for continuous variables and chi-square tests for categorical variables. Multivariable logistic regression models were used to examine the association between stressful life–event scores and probable PIU. Model 1 was unadjusted. Model 2 was adjusted for ethnicity and domicile. Model 3 was further adjusted for only-child status, accommodation type, living arrangement, current alcohol consumption, breakfast frequency, and outdoor activity time. Results were reported as odds ratios (ORs) with 95% CIs. Restricted cubic spline (RCS) analysis was used to examine potential nonlinearity in the association between stressful life–event scores and probable PIU. Candidate spline models with different knot specifications were compared using the Akaike Information Criterion and visual assessment of model fit. The final model included 4 knots located at the 5th, 35th, 65th, and 95th percentiles of the stressful life event score distribution, corresponding to scores of 0, 7, 21, and 69, respectively. A stressful life event score of 14 was used as the reference value. Overall and nonlinear associations were evaluated using likelihood ratio tests. Based on the 2 potential turning points identified from the spline curve, stressful life–event scores were further categorized as below 14, 14 to 60, and above 60. In categorical logistic regression analyses, the above 60 group was used as the reference category because the spline curve suggested a plateau beyond a score of 60, allowing lower-exposure groups to be compared with the high-exposure plateau group. The 95% CI for the proportion of participants meeting the screening criterion for probable PIU was calculated using the Wilson method. All statistical tests were 2-sided, and a *P* value less than .05 was considered statistically significant. Analyses were performed using R software (version 4.3.3; R Foundation for Statistical Computing).

### Ethical Considerations

This study was conducted in accordance with the Declaration of Helsinki and was approved by the Ethics Review Committee of Shanghai University of Medicine & Health Sciences (approval number: 2023-HXXM-01‐612401197903300537). Before the questionnaire link was distributed, trained investigators explained the study’s purpose, procedures, the voluntary nature of participation, and the right to withdraw; verbal informed consent was obtained from all participants. The questionnaire was completed anonymously, and no directly identifiable personal information was collected. No financial or material compensation was provided to participants. No identifiable participant images were included in this paper or supplementary materials.

## Results

### Participant Flow and Recruitment

**Figure 1.**
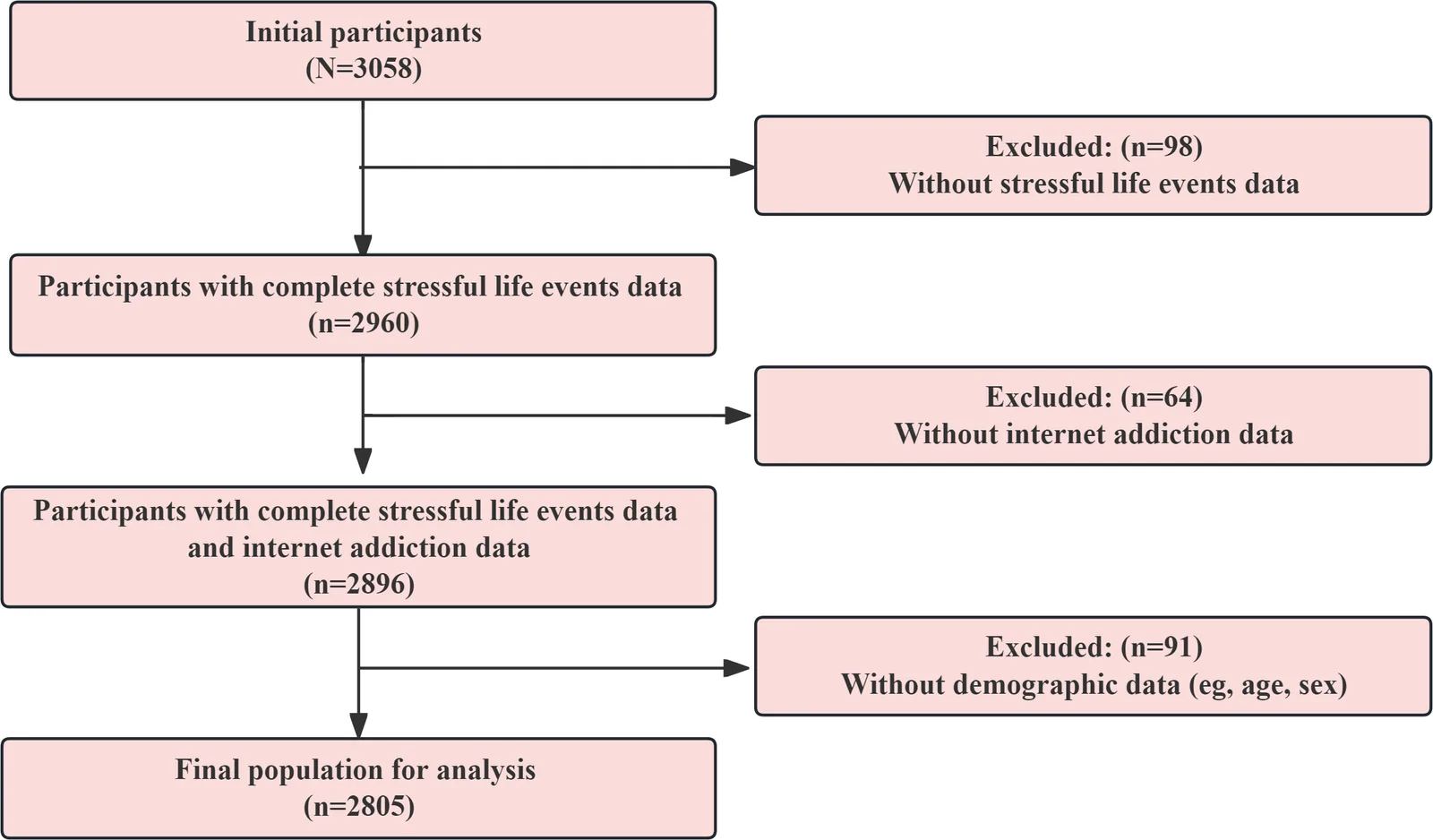
Participant flowchart for a cross-sectional survey of college students from 8 higher education institutions in eastern China from March 2025 to June 2025.

Participants were recruited between March 1, 2025, and June 30, 2025, from 8 higher education institutions in eastern China. A total of 3200 students were invited to participate, of whom 3058 submitted the questionnaire. After excluding 98 participants with missing data on stressful life events, 64 with missing IADQ data, and 91 with missing data for one or more prespecified covariates, 2805 participants were included in the complete-case analysis. The participant flow is presented in Figure 1.

### Participant Characteristics

Table 1 summarizes the baseline characteristics of the 2805 participants according to probable PIU status. Participants meeting the IADQ screening criterion for probable PIU reported substantially higher stressful life events scores than those not meeting the criterion (mean 30.26, SD 28.10 vs mean 17.75, SD 20.84; *P*<.001). Significant between-group differences were also observed in ethnicity, place of residence, only-child status, accommodation status, living arrangement, current alcohol consumption, breakfast frequency, and outdoor activity time. No significant differences were found in age, sex, or current smoking status.

**Table 1.** Baseline characteristics of participants according to probable problematic internet use (PIU) status^a^.

Characteristics	Overall (N=2805)	Probable PIU		*P* value
		Without (n=2369)	With (n=436)	
Age (y), mean (SD)	19.50 (1.40)	19.47 (1.36)	19.63 (1.60)	.12
Sex, n (%)				.30
Male	1209 (43.1)	1031 (85.3)	178 (14.7)	
Female	1596 (56.9)	1338 (83.8)	258 (16.2)	
Ethnicity, n (%)				.006
Han	1961 (69.9)	1632 (83.2)	329 (16.8)	
Others	844 (30.1)	737 (87.3)	107 (12.7)	
Domicile, n (%)				.01
City	1469 (52.4)	1217 (82.8)	252 (17.2)	
Country	1336 (47.6)	1152 (86.2)	184 (13.8)	
Only child, n (%)				.002
Yes	795 (28.3)	644 (81.0)	151 (18.9)	
No	2010 (71.7)	1725 (85.8)	285 (14.2)	
Accommodation status, n (%)				<.001
On-campus	2604 (92.8)	2217 (85.1)	387 (14.9)	
Off-campus	201 (7.2)	152 (75.6)	49 (24.4)	
Living arrangement, n (%)				<.001
With parents	2320 (82.7)	1996 (86.0)	324 (13.9)	
Father only	97 (3.5)	74 (76.3)	23 (23.7)	
Mother only	199 (7.1)	152 (76.4)	47 (23.6)	
Not with parents	189 (6.7)	147 (77.8)	42 (22.2)	
Current smoking, n (%)				.63
Yes	297 (10.6)	248 (83.5)	49 (16.5)	
No	2508 (89.4)	2121 (84.6)	387 (15.4)	
Current drinking, n (%)				<.001
Yes	683 (24.3)	537 (78.6)	146 (21.4)	
No	2122 (75.7)	1832 (86.3)	290 (13.7)	
Breakfast frequency, n (%)				.007
Daily	906 (32.3)	787 (86.9)	119 (13.1)	
Sometimes	1634 (58.3)	1372 (83.9)	262 (16.0)	
Never	265 (9.4)	210 (79.2)	55 (20.8)	
Outdoor activity time, n (%)				<.001
<1 h	795 (28.3)	636 (80)	159 (20)	
(1 h, 2 h)	1062 (37.9)	906 (85.3)	156 (14.7)	
(2 h, 3 h)	488 (17.4)	423 (86.7)	65 (13.3)	
≥3 h	460 (16.4)	404 (87.8)	56 (12.2)	
Stressful life events, mean (SD)	19.69 (22.58)	17.75 (20.84)	30.26 (28.10)	<.001

a Probable PIU was defined as an Internet Addiction Diagnostic Questionnaire (IADQ) score of 5 and above.

### Association Between Stressful Life Events and Probable PIU

**Figure 2.**
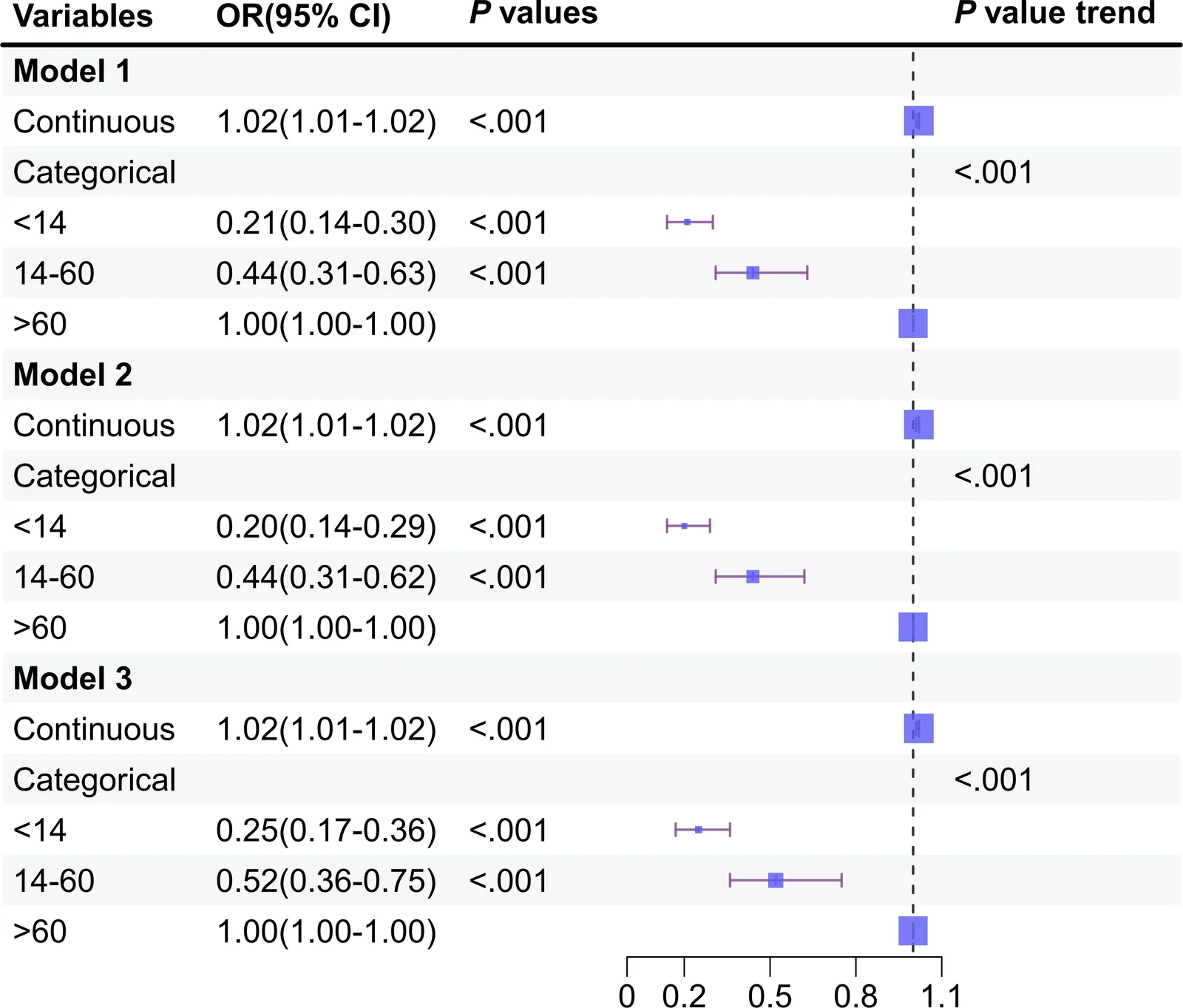
Multivariable logistic regression associations between stressful life–event scores and probable problematic internet use (PIU) among 2805 Chinese college students recruited from 8 higher education institutions in eastern China from March 2025 to June 2025. The original continuous variable of stressful life events was then transformed into 3 separate categories (<14, 14‐60, and >60) based on inflection points identified through restricted cubic splines (RCS). For the categorical analysis, the above 60 group was used as the reference category because the RCS curve suggested that the association reached a plateau beyond a score of 60, allowing the lower-exposure groups to be compared with the high-exposure plateau group. Model 1 was unadjusted. Model 2 was adjusted for ethnicity and domicile. Model 3 was adjusted for ethnicity, domicile, only child, accommodation status, living arrangement, current drinking, breakfast frequency, and outdoor activity time. Blue squares represent the odds ratio (OR) estimates, and blue horizontal lines represent the corresponding 95% CIs.

Figure 2 presents the association between stressful life events and probable PIU across 3 logistic regression models. When stressful life events scores were analyzed as a continuous variable, each one-point increase was associated with 2% higher odds of probable PIU in model 1 (OR 1.02, 95% CI 1.01‐1.02; *P*<.001). This association remained statistically significant in model 2 (OR 1.02, 95% CI 1.01‐1.02; *P*<.001) and model 3 (OR 1.02, 95% CI 1.01‐1.02; *P*<.001). Stressful life events scores were categorized as below 14, 14 to 60, and above 60 according to the inflection points identified by the RCS analysis, and participants with scores above 60 were used as the reference group. This group was selected as the reference because the spline curve suggested that the association reached a plateau beyond a score of 60, allowing the lower-exposure groups to be compared with the high-exposure plateau group. Compared with participants with scores above 60, participants with scores below 14 had lower odds of probable PIU in model 1 (OR 0.21, 95% CI 0.14‐0.30), model 2 (OR 0.20, 95% CI 0.14‐0.29), and model 3 (OR 0.25, 95% CI 0.17‐0.36). Similarly, compared with participants with scores above 60, participants with scores of 14 to 60 had lower odds of probable PIU in model 1 (OR 0.44, 95% CI 0.31‐0.63), model 2 (OR 0.44, 95% CI 0.31‐0.62), and model 3 (OR 0.52, 95% CI 0.36‐0.75). All categorical associations were statistically significant (*P*<.001).

### Nonlinear Association Between Stressful Life Events and Probable PIU

**Figure 3.**
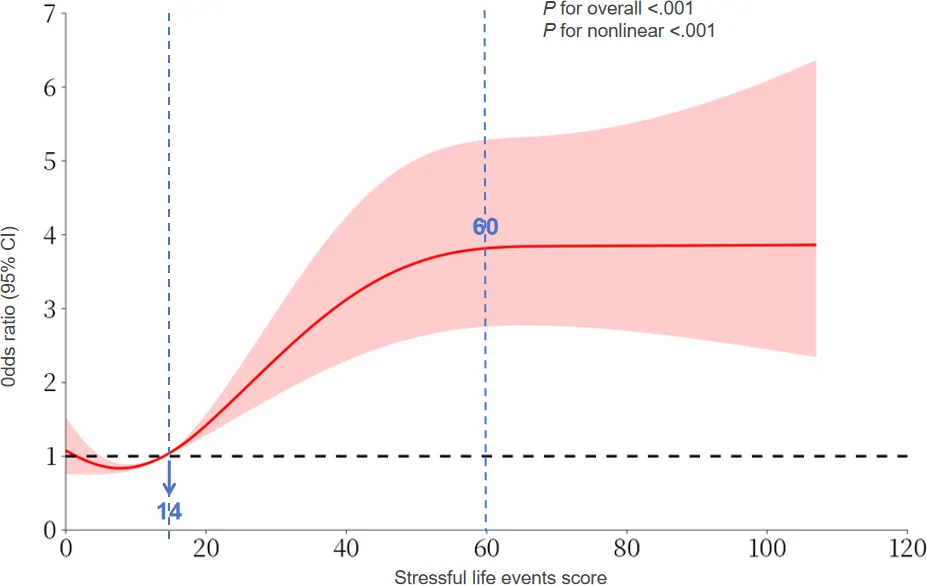
Restricted cubic spline analysis of the association between stressful life–event scores and probable problematic internet use (PIU) among college students from 8 higher education institutions in eastern China during March to June 2025. The solid red line represents the estimated odds ratio (OR), and the shaded area represents the 95% CI. The horizontal dashed line indicates an OR of 1.00. A stressful life event score of 14 was used as the reference value. The 4 knots were located at the 5th, 35th, 65th, and 95th percentiles, corresponding to scores of 0, 7, 21, and 69, respectively. The vertical dashed lines indicate the identified turning points at scores of 14 and 60. Participants with stressful life–event scores above 60 accounted for 5.9% (166/2805) of the sample. The relatively small number of participants in this range may have reduced the precision of the estimates and contributed to the wider CIs beyond a score of 60.

Figure 3 illustrates the dose-response association between stressful life events and probable PIU. An RCS model with 4 knots was used to examine potential nonlinearity in this association. The overall association was statistically significant (*P* value for overall <.001), with evidence of nonlinearity (*P* value for nonlinearity<.001). The odds of probable PIU changed little when stressful life events scores were below approximately 14. Between scores of approximately 14 and 60, the odds increased markedly with increasing stressful life events scores. Beyond approximately 60, the curve gradually plateaued, reaching about 3.9 times the odds observed at the reference value. Based on the turning points observed in the spline curve, stressful life events scores were subsequently categorized as below 14, 14 to 60, and above 60 for further analysis.

## Discussion

### Support for the Original Hypotheses

The present study examined the linear and nonlinear associations between stressful life–event scores and probable PIU among Chinese college students. The findings supported our hypotheses that higher stressful life–event scores would be associated with greater odds of probable PIU, and the association followed a nonlinear dose-response pattern. RCS analysis identified potential inflection points at approximately 14 and 60. These findings extend previous evidence based primarily on conventional linear models and provide a more nuanced characterization of the association between stressful life events and probable PIU [24,27,38].

### Similarity of Results

The positive association observed in the logistic regression models was consistent with a meta-analysis of Chinese adolescents and college students, which reported a significant correlation between stressful life events and PIU across 24 studies involving 21,944 participants (pooled *r*=0.34) [39]. Similar positive associations have also been reported in individual studies examining stressful or negative life events in relation to internet-related problematic behaviors among adolescents and college students [25-27]. Together, these findings suggest that the association is observable across different student populations and measures of stressful experiences, although the magnitude of the association may vary according to study design, sample characteristics, and the operational definition of PIU [25-27,39]. The persistence of the association across the 3 regression models suggests that the finding was not substantially altered by adjustment for the measured sociodemographic and lifestyle covariates, although residual confounding cannot be excluded. Importantly, the present study extends this literature by showing that the association may not be adequately represented by a strictly linear model, as the increase in the odds of probable PIU differed across levels of stressful life–event scores.

The proportion of participants meeting the IADQ screening criterion for probable PIU was 15.5% (436/2805), which was lower than the pooled estimate of 41.84% reported in a previous systematic review [8]. This discrepancy may reflect differences in assessment instruments, cutoff criteria, operational definitions, sampling procedures, regional and cultural contexts, and timing of data collection [8-11]. In particular, the present study used the IADQ with a prespecified cutoff of ≥5 to identify probable PIU rather than a clinical diagnosis, whereas studies included in previous reviews have used a variety of instruments and definitions of internet addiction or PIU [8]. Such methodological heterogeneity can substantially affect prevalence estimates and may partly explain the wide variation reported across university populations [8-11]. Therefore, prevalence estimates derived from different instruments and study populations should not be interpreted as directly comparable [8-11]. The present estimate should consequently be understood as the proportion of participants meeting the screening criterion for probable PIU within this specific sample rather than as an estimate of the prevalence of clinically diagnosed internet addiction among Chinese college students.

Probable PIU was also associated with several sociodemographic and lifestyle characteristics. It was more common among urban residents, only children, students living off campus, those not living with both parents, current drinkers, students with less frequent breakfast consumption, and those reporting shorter outdoor activity time. Differences in psychosocial stress, family environment, personal autonomy, offline social interaction, socioeconomic circumstances, and access to digital devices may partly explain these associations; however, these mechanisms were not directly assessed and remain hypothetical [25]. Reduced parental supervision may partly account for the association with off-campus living and nonparental living arrangements [25,28], although differences in financial resources, autonomy, and social networks may also contribute [40,41]. The associations with alcohol consumption, breakfast frequency, and outdoor activity are consistent with previous evidence linking PIU to less healthy lifestyle patterns [13,37]. These lifestyle characteristics may also coexist within broader patterns of health behavior rather than operate independently [13,37]. Nevertheless, the direction of these associations cannot be determined because probable PIU may contribute to disrupted health behaviors, while such behaviors may also be associated with greater vulnerability to PIU. Accordingly, these secondary associations should be interpreted primarily as descriptive correlates rather than as evidence of causal risk factors.

### Interpretation

The findings may also be interpreted within the framework of stress and coping theory, which proposes that individuals use different strategies to regulate emotions arising from stressful experiences [42]. The internet may provide college students with an accessible means of temporarily disengaging from stressful circumstances, regulating negative emotions, and maintaining social connectedness through online activities such as social media use [43,44]. Avoidant coping strategies, including distraction, denial, and withdrawal from offline problems, may increase reliance on the internet for emotional escape [44]. In contrast, more adaptive coping strategies, such as problem solving and seeking social support, may reduce reliance on internet use for emotional escape [42,45].

Persistent use of the internet as an avoidant coping strategy may contribute to poorly controlled or compulsive use and increase the likelihood of meeting the screening criterion for probable PIU [46]. Recent evidence also suggests that experiential avoidance and psychological distress may help explain how negative life events are translated into problematic patterns of digital media use [47]. From this perspective, stressful experiences may not directly lead to probable PIU; rather, their effects may depend partly on how students appraise stressful situations and the coping strategies available to them [42,44,47]. However, coping strategies and psychological distress were not directly assessed in this study, and their potential mediating or moderating role requires further investigation.

The RCS analysis further indicated that the association was nonlinear. At relatively low stressful life–event scores, the estimated association was weak and remained close to the reference level. Mild stress may remain manageable through adaptive coping resources and health-promoting behaviors, including physical activity [42,48,49]. Students exposed to relatively few or less impactful stressful events may therefore retain greater coping resources for managing stress without substantially increasing their reliance on the internet as a coping mechanism [42,48,49]. As stressful life–event scores increased, the association with probable PIU became substantially stronger, which may reflect the progressive depletion of coping resources and greater reliance on the internet as a means of emotional avoidance [47]. This interpretation is compatible with evidence linking higher levels of academic or life stress with more problematic patterns of internet use [39,46]. As cumulative stress increases, repeated exposure to negative experiences may place greater demands on self-regulation and increase the attractiveness of immediately accessible online activities for distraction, emotional regulation, or temporary escape [43,44,47]. These mechanisms provide a plausible explanation for the steeper increase in the estimated odds of probable PIU between scores of approximately 14 and 60, although they were not directly tested in the present study.

At very high stressful life–event scores, the estimated association appeared to plateau. One possible explanation is that severe cumulative stress may be accompanied by emotional exhaustion or co-occurring psychological problems, such as depression and anxiety, which may alter the relationship between stress and internet use [24]. At these levels, additional stressful experiences may produce relatively smaller changes in internet-related behavior because psychological distress and maladaptive coping may already be pronounced [24,39,47]. Alternatively, severe stress may be associated with multiple competing behavioral and psychological consequences, such that internet use represents only one of several possible responses to cumulative adversity [24,39]. However, relatively few participants had scores above 60, and the wider CI in this range indicate reduced statistical precision. The observed flattening of the spline may therefore represent a true saturation pattern, a consequence of the relatively small number of highly exposed participants, or a combination of both. Therefore, the apparent plateau should be interpreted cautiously and confirmed in studies with larger numbers of participants experiencing very high levels of stressful life events. The values of approximately 14 and 60 should likewise be regarded as exploratory data-derived inflection points rather than biological thresholds or clinically validated cutoffs.

### Strengths and Limitations

This study has several strengths. First, the relatively large multicenter sample improved the statistical precision of the estimates and provided sufficient observations across a broad range of stressful life–event scores. Second, the combined use of conventional logistic regression and RCS analysis allowed the association to be examined without assuming a strictly linear dose-response pattern. This approach provided a more detailed characterization of changes in the odds of probable PIU across different levels of stressful life events. Third, the analyses incorporated multiple sociodemographic and lifestyle covariates, thereby reducing, although not eliminating, the potential influence of measured confounding. Fourth, stressful life events and probable PIU were assessed using established instruments with good internal consistency in the present sample. The inclusion of students from different types of higher education institutions also increased the diversity of the study sample within the surveyed region.

Several limitations should be acknowledged. First, the cross-sectional design precludes conclusions regarding temporality or causality, and reverse causality remains possible. In addition, because data collection extended through the end of June and academic calendars varied across institutions, some participants may have completed the survey during examination periods, potentially affecting reported stress levels or internet-use patterns. Second, stressful life events, probable PIU, and lifestyle characteristics were self-reported and may therefore be subject to recall bias, social desirability bias, and measurement error. The ASLEC does not capture the precise timing, frequency, duration, or recurrence of stressful events, whereas the IADQ does not provide objective information on screen time or specific types of internet use. The IADQ is a screening instrument rather than a clinical diagnostic measure. Third, convenience sampling and recruitment through class-based WeChat groups may have introduced selection bias. Fourth, several potentially relevant socioeconomic and academic factors were not assessed, and residual confounding cannot be excluded. Moreover, 253 of the 3058 (8.3%) respondents were excluded because of missing data. Because the data were not missing completely at random, complete-case analysis may have introduced additional selection bias, and residual bias related to missing data cannot be excluded. Future studies should use longitudinal designs, more detailed measures of stressful life events and coping, objective indicators of internet use, and more diverse samples.

### Generalizability

The findings should primarily be generalized to college students attending institutions similar to those included in this study. Convenience sampling was used, and participants were recruited from 8 higher education institutions in Shanghai, Jiangsu, and Zhejiang through class-based WeChat groups. The participating institutions were selected partly on the basis of existing collaborations and willingness to participate. Nevertheless, the inclusion of comprehensive universities, medical universities, and vocational colleges provided some diversity in educational settings and suggested that the observed association may be relevant to students studying in different types of higher education institutions within the surveyed region. The relatively large sample also included students with a broad range of stressful life–event scores, thereby strengthening the relevance of the observed dose-response pattern within comparable university populations.

At the same time, external validity should be considered in relation to geographic, educational, cultural, and measurement contexts. The sample may not be representative of all college students in China, particularly students in other geographic, cultural, or educational settings. Accordingly, caution is warranted when extrapolating the findings to students in other geographic, cultural, or educational contexts. Similarly, the findings should not be assumed to apply directly to adolescents, working young adults, or populations outside China because these groups were not represented in the present sample. The use of self-report instruments and the specific IADQ cutoff may also limit comparability with studies using other measures or diagnostic frameworks [8-11]. Furthermore, associations observed using generalized PIU measures may not necessarily be identical to those obtained using measures of specific problematic online behaviors, such as problematic gaming, social media use, or smartphone use [11,25,36,40]. Replication using probability-based sampling, more geographically diverse populations, and alternative validated measures of generalized and activity-specific PIU is needed before the identified nonlinear pattern and potential inflection points can be considered broadly generalizable.

### Implications

The potential inflection points identified at approximately 14 and 60 may have practical implications, although they should be regarded as exploratory statistical values rather than validated clinical cutoffs. At the individual level, the nonlinear pattern suggests that stressful life events may be considered as one component of a broader psychosocial risk assessment rather than an isolated screening criterion [50]. Because the estimated odds of probable PIU increased more markedly across the middle range of stressful life–event scores, students experiencing accumulating stress may represent an important group for early preventive support [50]. Students with moderately elevated stressful life–event scores may benefit from stress-management and coping-skills interventions, peer support, accessible psychological counseling, and opportunities for regular physical activity [48,50,51]. Such approaches may strengthen adaptive coping resources and provide alternatives to relying on online activities primarily for emotional escape or avoidance [44,45,50].

Students experiencing very high levels of stressful life events may warrant a broader assessment of psychological distress, functional impairment, coping resources, and the severity and consequences of PIU [12,14,15,50]. The apparent plateau above a score of approximately 60 should not be interpreted as indicating that additional stress is harmless or that students in this range are at lower clinical priority. Rather, very high cumulative stress may signal broader psychological vulnerability and the need for multidimensional assessment and support [14,50,51]. At the institutional level, universities could consider incorporating assessment of stressful life events into broader mental health services and establishing tiered pathways linking early identification, stress-management education, peer support, psychological counseling, and professional referral [50]. Such screening would be most appropriate when combined with assessment of psychological distress, functioning, and internet-related impairment rather than using the ASLEC score alone to determine intervention [14,15,51]. Furthermore, the potential inflection points identified in this study should not currently be used as fixed thresholds for clinical decision-making until they have been replicated and externally validated.

Future studies should use longitudinal designs to clarify temporal relationships and examine whether stressful life events predict the onset or persistence of probable PIU. More detailed measures are also needed to distinguish acute, recurrent, and chronic stressors and to assess the timing, duration, and frequency of stressful experiences. Future research should additionally examine coping strategies, psychological distress, and specific internet activities as possible mediators or moderators. The proposed nonlinear pattern and potential inflection points should be replicated in larger and more diverse samples.

### Conclusions

This study is innovative in applying RCS analysis to characterize the nonlinear dose-response association between stressful life–event scores and probable PIU among Chinese college students. Unlike previous studies that relied primarily on conventional linear models, our findings identified potential inflection points at approximately 14 and 60 and provided a more detailed description of how the odds of probable PIU varied across different levels of stressful life events. These findings extend the existing evidence and may provide preliminary information for the development of tiered screening, stress-management support, and referral pathways in university mental health services. However, the identified values should be regarded as exploratory rather than validated clinical cutoffs, and longitudinal and intervention studies are needed to confirm their practical use.

## Abbreviations

ASLEC: Adolescent Self-Rating Life Events Checklist
IADQ: Internet Addiction Diagnostic Questionnaire
MCAR: Missing Completely at Random
OR: odds ratio
PIU: problematic internet use
RCS: restricted cubic spline
